# A structured approach to improving patient safety: Lessons from a public safety-net system

**DOI:** 10.1186/1754-9493-5-32

**Published:** 2011-12-01

**Authors:** Philip S Mehler, Christopher B Colwell, Philip F Stahel

**Affiliations:** 1 Department of Patient Safety and Quality, Denver Health Medical Center, 777 Bannock Street, Denver, Denver, CO 80204; 2 Department of Internal Medicine, Denver Health Medical Center, 777 Bannock Street, Denver, Denver, CO 80204; 3 Department of Emergency Medicine, and Denver Paramedic Division, Denver Health Medical Center, 777 Bannock Street, Denver, CO 80204, USA; 4 Denver Paramedic Division, Denver Health Medical Center, 777 Bannock Street, Denver, CO 80204, USA; 5 Department of Orthopaedic Surgery, Denver Health Medical Center, 777 Bannock Street, Denver, CO 80204, USA; 6 Department of Neurosurgery, University of Colorado, School of Medicine, Aurora, CO 80045, USA

## Introduction

Denver Health is a public, academic health system, a model integrated system of care, and Colorado's principal safety-net institution. It serves a third of Denver's citizens including the most vulnerable, with 75% of its patients having incomes below 185 percent of the federal poverty level, two-thirds being ethnic minorities and almost one-half are uninsured. These patient characteristics embody health care disparities which typically impede the intended outcomes of a system's quality and safety interventions [1]. In reality, however, it was precisely these challenges which inspired Denver Health's leadership to embark upon a relentless journey towards quality improvement seven years ago [2]. Specifically, in consideration of a safety-nets limited resources in the setting of a population of socially disadvantaged and clinically complex patients, Denver Health's quality program was impelled to focus on creating programs to manage high-risk and high-opportunity clinical situations. Although Denver Health's renewed structured approach to quality and safety began seven years ago, a number of foundational elements were already in place including most importantly an integrated health care system which provides seamless continuity of care in the setting of a system of care which is staffed by an employed physician medical staff. This employed-physician model promotes alignment of goals across the enterprise and helps implement new quality and safety interventions [2].

### Quality and safety interventions

Some of Denver Health's recent programs to manage high-risk/high-opportunity areas include our unique rapid response system to prevent "failure to rescue" [3,4]. Indeed, a recent study of postoperative mortality stressed "failure to rescue," rather than the number of complications, as the key variable in explaining differences in mortality rates cross hospitals [5]. Using our "clinical triggers" to identify clinical deterioration, we were able to reduce our cardiopulmonary arrest rate and the number of patients requiring transfer back to an intensive care unit within 48 hours after having been previously transferred to a hospital ward [3].

In addition, we instituted hospitalist co-management for all patients on the orthopedic service, patients on low-volume inpatient surgical subspecialty services and patients on the psychiatry service with significant medical comorbidities. Moreover, a formal and robust antibiotic stewardship program was established. This approach spawned new programs including mandatory infectious disease consultation for certain serious infections, concurrent feedback to a prescribing team when multiple antibiotics were used for the same patient and formal infectious disease consultant rounds with intensive care unit teams. As a result Denver Health's antibacterial drug use was the lowest of thirty-five U.S. academic health centers reporting through the University Health System Consortium (UHC) [6]. Moreover, proper treatment of infections has increased - and adverse consequences from illness have decreased - for the highly virulent and prevalent staphylococcus aureus bacteremia [7]. Another high-risk condition in hospitalized patients, and the leading cause of potentially preventable death, is represented by venous thromboembolism (VTE). By designing and implementing an evidence-based risk-assessment tool and clinical practice guideline, embedded into admission order sets in the computerized physician order system (CPOE), the compliance with the VTE prophylaxis guideline was drastically increased [8]. Denver Health's performance in preventing venous thromboembolism climbed to the top 10 percent nationwide [8].

### Quality Assurance (QA) process in Orthopaedics

Surgical patients remain highly susceptible to preventable perioperative complications, despite the nationwide implementation of standardized patient safety protocols in recent years. Preventable adverse occurrences include so-called "never events", such as wrong-patient and wrong-site surgery [9,10]. Recent publications emphasize the fact that our current patient safety protocols are indeed not safe in protecting our patients from suffering unintended and preventable harm [11-13]. New strategies to improve patient safety in surgery include the implementation of defined surgical safety checklists, standardized "readbacks" to improve communication in perioperative services, and medical team training programs [14-18].

Disclosing and reporting of medical errors is compelling beyond a doubt from a moral, ethical, and scientific perspective, and therefore represents a basic tenet for improving patient safety. Underreporting of surgical complications creates a gap of information which may otherwise help prevent the recurrence of a similar adverse event. The Department of Orthopaedics at Denver Health implemented a new Quality Assurance (QA) process in 2007 [19]. This new QA protocol was designed to lower the threshold of reporting all perceived complications, "near-misses", and "no-harm events", mandating a standardized peer-review of all reported occurrences in a "real-time" fashion, and relies on the following three cornerstones:

*1. Anonymous "real-time" reporting of any suspected adverse occurrence, including "near miss" and "no harm" events, by any member of the surgical team. Occurrences are reported to an independent nurse provider in charge of managing the adverse event database. A "no fault" policy for reporting occurrences is encouraged with strong support from the department leadership*.

*2. Peer-review of each reported event at a weekly QA conference, using a standardized case review form, in the presence of the responsible attending surgeon and at least two additional faculty staff members who were not involved in the occurrence*.

*3. Corrective action is defined for each reviewed case, if deemed necessary during the peer-review process. Each closed case is prospectively entered into a departmental QA database. All team members involved in the adverse occurrence are notified about the final assessment of the peer-review process*.

Within two years of implementation of this new QA process, the median rate of reported occurrences increased more than 6-fold from 1.7 to 11.1 per 100 surgical procedures [19]. Similarly, the overall complication rate for the entire Department of Orthopaedics at Denver Health increased almost 5-fold, from 1.4% to 6.7%. These data emphasize the "double-edged sword" aspect of reporting adverse events: The reported 5-fold increase in complications within the department likely reflects the improved open and more honest reporting format and critical peer-review of each reported occurrence, rather than a decreased quality of care. And herein lies the paradox: If the parameters of "reported adverse events" and "incidence of complications" were used as a measure of institutional quality, the facility would be penalized for its improved surveillance and educational process. The thorough reporting and peer-review of surgical errors creates a new dilemma for the practicing surgeon: an increased quality of reporting leads to an increased complication rate, thus affecting the individual surgeon's professional track record and the respective institution's ranking among peers. Until legislation provides legal protection for medical error disclosure and analysis, we continue to rely on the limited and anecdotal reporting of medical errors and surgical complications in the peer-reviewed biomedical literature [20,21].

### Coordinated management of high risk patients

As a part of Denver Health's integrated system, the Emergency Department (ED) has identified a number of areas where coordination of care may benefit quality of care and outcomes measures. There was recognition that variance in approaches to care for several common diagnoses may adversely impact quality in terms of percentage of Intensive Care Unit (ICU) admissions, length of stays, and outcomes. In particular, acute alcohol withdrawal, sepsis, and diabetic ketoacidosis were identified as having variable ICU admission rates as well as a high rate of change in management after the patient had transitioned from the Emergency Department to the ICU. In particular, management was changed after transition to the ICU in more than 33% of patients with these admitting diagnoses. In response, a group consisting of emergency physicians, intensive care specialists, infectious disease specialist, and endocrinologists convened to create guidelines for the management of these specific entities to ensure a coordinated and standardized approach across the institution for the initial management of these patients. Outcomes measures will include ICU admission rates, intubation rates, length of stay in the ICU and hospital, as well as return visits to the Emergency Department for all three identified entities to determine the true impact of this coordination. The goal will be to improve institutional consistency in the management of disease processes in order to reduce waste and redundancy. Moving forward, the identification of evidenced based quality measures that reach across specialties and encouraging a multispecialty approach to the management of these entities from the initial point of care should enhance quality as well as the patient experience.

The evaluation of ED quality of care has been hampered by the absence of consensus on appropriate measures [22]. Some investigators have used a Modified-Delphi process to identify specific condition-outcome pairs linking quality of care to specific outcomes [23]. They identified asthma, pneumonia, acute myocardial infarction, deep vein thrombosis/pulmonary embolism, chest pain, minor head trauma, and ankle/foot trauma as clinical conditions for which indicators could be identified and measured to assess quality of care within the ED. Another group looking specifically at pediatric emergency care found 405 performance measures that could potentially be used, but that these measures lack a systematic and comprehensive approach to evaluate the quality of care provided [24]. Future efforts will need to focus on validating condition-outcome pairs that are managed by multiple specialties in order to support efforts towards benchmarking and quality improvement following the structure-process-outcome that has been used to guide clinical quality improvement initiatives [25,26].

## Conclusion

As a result of the structured approach to quality and safety at our safety-net system, and ongoing efforts in areas wherein improvement opportunities exist, Denver Health was ranked first of all academic medical centers with the lowest observed-to-expected (O/E) mortality ratio in the 2011 UHC Annual Quality and Accountability Aggregate Score. In addition, Denver Health's trauma mortality rate is the lowest of all large level 1 trauma centers in the UHC database and also in the Colorado's Department of Public Health's most current trauma registry. Denver Health with its integrated system of care, employed physician model, and commitment to transparency, bolstered by a strong health information technology infrastructure, has irrefutably demonstrated that excellent care quality and patient safety can be successfully advanced within healthcare institutions even when challenged by limited resources and socially disadvantaged and complex patients.

## Competing interests

All authors are employed by Denver Health. PSM is the chief medical officer, CBC is the director of Emergency Medicine, and PFS is the Director of Orthopaedics at Denver Health. The authors declare no other competing interests related to this editorial.

## Authors' contributions

All authors contributed equally to drafting this editorial, and read and approved the final version of the manuscript.
